# HOXA5 Targets ABCD1 and IRF7 to Regulate the Proliferation and Differentiation of Goat Intramuscular Preadipocytes

**DOI:** 10.3390/ani16213537

**Published:** 2026-11-09

**Authors:** Dingshuang Chen, Xuening Li, Hongwei Ding, Jintao Zhong, Nan Zhao, Yanyan Li, Wei Liu, Youli Wang, Jiani Xing, Yaqiu Lin

**Affiliations:** 1 College of Animal Science and Veterinary, Southwest Minzu University, Chengdu 610041, China; 2 Key Laboratory of Qinghai-Tibetan Plateau Animal Genetic Resource Reservation and Utilization, Ministry of Education, Southwest Minzu University, Chengdu 610041, China; 3 Key Laboratory of Sichuan Province for Qinghai-Tibetan Plateau Animal Genetic Resource Reservation and Exploitation, Southwest Minzu University, Chengdu 610041, China

**Keywords:** HOXA5, lipid accumulation, IRF7, ABCD1, RNA-Seq

## Abstract

Intramuscular fat (IMF) is crucial for goat meat quality, being influenced by preadipocyte proliferation and differentiation. Although the developmental transcription factor HOXA5 is associated with adipose tissue development and lipid deposition in mice, its role in goat adipogenesis remains unclear. This study investigated HOXA5’s role in regulating IMF lipid deposition in goats. Regarding differentiation, HOXA5 overexpression reduced mRNA and protein levels of differentiation genes, including CEBP/α and CEBP/β. Additionally, Oil Red O and Bodipy staining demonstrated a decrease in lipid accumulation in the HOXA5 overexpression group. Conversely, these phenotypes showed opposite trends in the HOXA5 knockdown group. Regarding proliferation, HOXA5 overexpression upregulated mRNA levels of proliferation genes, enhanced cell vitality, and increased the proportion of EdU-positive cells, while HOXA5 knockdown inhibited these phenotypes. Mechanistically, *IRF7* and *ABCD1* were identified as the target genes of HOXA5 by RNA-Seq and dual luciferase reporter gene assay. Functional analyses further showed that IRF7 promoted both proliferation and adipogenic differentiation, whereas ABCD1 inhibited both processes. Rescue experiments supported the involvement of IRF7 and ABCD1 in HOXA5-induced promotion of proliferation and inhibition of adipogenic differentiation. These results highlight HOXA5’s critical regulatory role in goat intramuscular adipocyte lipid deposition, offering novel insights into the underlying molecular mechanisms.

## Simple Summary

Intramuscular fat (IMF) is an important factor affecting goat meat quality, but the molecular mechanisms regulating IMF deposition remain unclear. In this study, we investigated the role of HOXA5 in goat intramuscular adipocytes. HOXA5 promoted adipocyte proliferation but inhibited differentiation and lipid accumulation. RNA-Seq and dual-luciferase assays identified IRF7 and ABCD1 as potential target genes of HOXA5. These findings reveal a novel role of HOXA5 in regulating goat intramuscular fat deposition and provide new insights into the molecular mechanisms underlying goat meat quality.

## 1. Introduction

Mutton is widely consumed because of its delicious taste and rich nutritional value. Intramuscular fat (IMF) is a crucial factor affecting meat quality [1]. IMF, a type of fat deposited between muscle fibers, has a negative impact on human health and is strongly associated with obesity and insulin resistance, but has a positive impact on meat quality in goats, and is associated with meat tenderness, flavor and juiciness [2,3,4,5,6]. Mutton with a high IMF content usually has higher consumption preference. Therefore improving meat quality by increasing the content of IMF has become an important topic in academic research [7]. Multiple processes are involved in the formation of IMF, mainly including the differentiation and proliferation of intramuscular preadipocytes [8]. These processes are also subject to intricate regulation by an array of genes. For example, the differentiation of intramuscular preadipocytes is modulated by key cell differentiation regulators, primarily encompassing the peroxisome proliferator-activated receptors (PPARs) family, the CCAAT-enhancer-binding protein (C/EBPs) family, and the sterol regulatory element-binding protein (SREBP) family [9]. Similarly, the proliferation of intramuscular preadipocytes is influenced by several cell cycle regulatory factors, including cyclin-dependent kinase inhibitors (CKIs) and cyclin-dependent kinases (CDKs) [10]. In addition, several key transcription factors (TFs) have been identified as being directly or indirectly involved in the formation of IMF; however, numerous significant TFs have yet to be reported.

Homeobox A5 (HOXA5), a transcription factor belonging to the Homeobox (HOX) gene family, is located on chromosome 7p15.2. It contains a 183-base-pair DNA sequence known as the Homeobox, which encodes a highly conserved homeodomain [11,12]. HOXA5 regulates cell differentiation, proliferation, and embryonic development by binding to the promoters of numerous downstream target genes and modulating their expression [13]. Previously published evidence suggests that HOXA5 plays a crucial role in skeletal formation during development [14]. Subsequent research has demonstrated that aberrant expression of HOXA5 can result in organ abnormalities and dysfunction, influencing biological processes such as proliferation, differentiation, invasion, and apoptosis across various cancer types [15,16,17,18,19,20]. Beyond its established roles in development and oncogenesis, accumulating evidence indicates that HOXA5 is also implicated in the regulation of adipogenesis [21]. In mouse precursor adipocytes, the gain or loss of HOXA5 function respectively promotes or inhibits adipogenesis [22,23]. Furthermore, in human subcutaneous adipose tissues, depletion of HOXA5 impairs adipocyte differentiation, leading to reduced lipid accumulation and decreased expression of key adipogenic markers [21]. While our prior research has demonstrated that HOXA5 facilitates subcutaneous adipocyte differentiation and suppresses subcutaneous adipocyte proliferation in goats [24], the role of HOXA5 in intramuscular lipid deposition in goats remains to be elucidated.

Interferon regulatory factor 7 (IRF7) is a multifunctional transcription factor within the interferon regulatory factor (IRF) family [25]. IRF7 is pivotal in host defense mechanisms against a range of bacterial and viral infections by modulating the transcription of interferons (IFNs) and the expression of IFN-stimulated genes (ISGs) [26]. Recent studies have elucidated additional roles for IRF7, demonstrating its involvement in both pro-inflammatory and anti-inflammatory processes across various inflammatory diseases [27,28]. These functions are mediated through diverse signaling pathways, including the NF-κB signaling pathway and autophagy activation. In addition to its role in the regulation of inflammation, IRF7 is implicated in the regulation of cell apoptosis, differentiation, and proliferation [29,30,31,32]. Consequently, IRF7 is a significant factor in tumorigenesis and metastasis. Studies have demonstrated a marked increase in the expression levels of IRF7 in the adipose tissue of obese children [33]. And IRF7 may contribute to the pathogenesis of obesity in murine models through the regulation of the proinflammatory adipokine MCP-1 [34]. These findings suggest that IRF7 may play a role in adipogenesis.

ATP-binding cassette (ABC) transporters constitute the largest superfamily of membrane-binding proteins, facilitating the transport of a diverse array of substrates across both the outer and inner cellular membranes [35,36]. ABCD1 is a member of the ABC transporter subfamily D, which plays a crucial role in the transport of various fatty acid coenzyme A derivatives and very long chain fatty acids (VLCFAs) to the peroxisome. It is vital for peroxisomal metabolism and lipid homeostasis [37]. Dysfunction of human ABCD1 has been implicated in neurodegenerative disorders, including X-linked adrenoleukodystrophy (X-ALD) [38]. Currently, research on ABCD1 is focused on X-ALD, with few studies investigating its role in adipogenesis.

In the present study, we investigated the effects and underlying cellular mechanisms of HOXA5 in goat intramuscular preadipocyte formation. The results reveal that HOXA5 could enhance goat intramuscular preadipocyte proliferation while suppressing differentiation. Mechanistically, HOXA5 mediates this process by targeting two downstream genes, *IRF7* and *ABCD1*, which exhibit opposite functions. IRF7 acts as a positive regulator of both proliferation and differentiation in goat intramuscular preadipocytes, whereas ABCD1 functions as a negative modulator of these processes. Altogether, HOXA5 exerts a unique dual regulatory role in the proliferation and differentiation of goat intramuscular adipocytes by modulating two functionally opposing target genes, *IRF7* and *ABCD1*, thus showing an inhibitory effect on IMF deposition.

## 2. Materials and Methods

### 2.1. Ethics Statement

Three one-week-old healthy male Jianzhou big-eared goats were used for preadipocyte isolation and culture. All animal procedures followed the guidelines established by the Institutional Animal Care and Use Committee at Southwest Minzu University, China (Project identification code: SMU-202601244).

### 2.2. Isolation, Culture and Differentiation of Goat Intramuscular Preadipocyte

The isolation of goat intramuscular preadipocytes (hereafter referred to as GIPCs) was performed as previously established in our laboratory [39]. In short, the longissimus dorsi muscle from three one-week-old Jianzhou big-eared goats was washed in 1 × PBS, and the fascia and the connective tissue were gently removed with sterile forceps. Then, the longissimus dorsi muscle was minced and digested in a solution containing 0.25% Collagenase II (Sigma, St. Louis, MO, USA) for 2 h at 37 °C. The cell suspension was passed through 200-mesh and 400-mesh filters in sequence and then centrifuged at 1500 rpm for 5 min. Then, RBC lysis solution was used to remove the blood cells, and the cell suspension was centrifuged at 1500 rpm for 5 min again to obtain the cell pellet. Finally, the cell pellet from each animal was separately resuspended in DMEM/F-12 (Gibco, Carlsbad, CA, USA) supplemented with 1% penicillin–streptomycin (Gibco, Carlsbad, CA, USA) and 10% fetal bovine serum (Gemini, Calabasas, CA, USA) and seeded into separate 100 mm culture dishes. Cells from the three animals were maintained as three independent primary cell cultures without pooling. For adipocyte differentiation, GIPCs were seeded into 6-well plates at a density of 8 × 10^4^ cells and induced with adipogenesis induction medium containing 50 μmol·L^−1^ oleic acid for 48 h.

### 2.3. Plasmid Construction and Transfection

The overexpression plasmid of HOXA5 was constructed by inserting the amplified *HOXA5* cDNA (GenBank accession No. MZ004987) fragments into the pEGFP-N1 vector (the primers for amplification of *HOXA5* cDNA are listed in Table 1). The IRF7 and ABCD1 overexpression plasmids were constructed using the same procedure, with their respective coding sequences inserted into the pEGFP-N1 vector.

Small interfering RNAs against HOXA5 were synthesized by Invitrogen (Invitrogen, Carlsbad, CA, USA). The sequence targeting HOXA5 was as follows: 5′-UGAAUUGCUCGCUCACGGAACUAUGdTdT-3′. The sequence of the negative control SiRNA was as follows: 5′-UUCUCCGAACGUGUCACGUdTdT-3′.

CRISPR/Cas9 plasmids targeting IRF7 and ABCD1 were constructed using the PX459 vector. Target-specific sgRNAs were designed, and the corresponding complementary DNA oligonucleotides were synthesized by Sangon Biotech (Shanghai, China). The oligonucleotides were annealed and ligated into the BbsI-digested PX459 vector. The ligation products were transformed into competent Escherichia coli cells, and individual colonies were cultured for plasmid extraction. Correct insertion of the target sequences was confirmed by sequencing at Sangon Biotech.

TurboFect Transfection Reagent (Thermo Fisher Scientific, Waltham, MA, USA) was used for all transfection experiments according to the instructions. Briefly, using a 6-well plate as an example, 2 μg of plasmid or 6 μL of siRNA (20 μM) was transfected into GIPCs when the cell density reached about 80%. The transfection medium was replaced with fresh complete medium for further culture after 12 h. The transfected GIPCs were collected for subsequent experiments at 48 h post-transfection.

### 2.4. EdU Staining

At 48 h after transfection, GIPCs were incubated with a 50 mM EdU working solution (Solaibao, Beijing, China) for 2 h, fixed with 4% paraformaldehyde, and stained according to the manufacturer’s protocol. Lastly, images of EdU-stained samples were acquired using a microscope (OLYMPUS IX71, Tokyo, Japan).

### 2.5. Oil Red O Staining and Bodipy Staining

The GIPCs’ differentiation was evaluated by Oil Red O staining and Bodipy staining, as described in our earlier study [40]. Briefly, GIPCs were fixed with 4% paraformaldehyde for 15 min at room temperature after being washed twice with PBS. Cells were incubated with Oil Red O (Solarbio, Beijing, China) for 20 min. After that, cells were washed twice with PBS. For Bodipy staining, the fixed cells were stained with a Bodipy working solution (1 μg/mL, Thermo Fisher Scientific, Waltham, MA, USA) in the dark for 20 min. Cells were then treated with DAPI working solution (5 μg/mL, Solarbio, Beijing, China) to stain the nuclei. Images were taken with an OLYMPUS IX7 fluorescence microscope after samples were washed twice with PBS. After microscopic observation, the Oil Red O-stained cells were incubated with 1 mL per well of isopropanol for 30 min to quantify lipid droplets.

### 2.6. Cell Cycle Assay

At 48 h post-transfection, GIPCs were digested with 0.25% trypsin and collected into centrifuge tubes. Cells were then washed twice with pre-cold PBS and fixed with 70% ethanol at 4 °C overnight. Finally, GIPCs were stained using a Cell Cycle Assay kit (Nanjing Keygen, Nanjing, China) and analyzed with a flow cytometer (BD Biosciences, Franklin Lakes, NJ, USA).

### 2.7. MTT Analysis and Crystal Violet Staining

The proliferation of GIPCs was assessed by the MTT experiment and crystal violet staining. For the MTT experiment, GIPCs (1 × 10^3^/well) were seeded into 96-well plates. The transfected cells were continuously cultured in 96-well plates, and 10 µL of MTT (5 mg/mL) was added at 0, 24, 48 and 72 h. After 4 h, dimethyl sulfoxide (150 µL/well) was added, and the absorbance at 490 nm was detected using a microplate reader.

### 2.8. Western Blot Analysis

The total protein of GIPCs was extracted using RIPA (Sangon, Shanghai, China), and a BCA protein assay kit (Biosharp, Beijing, China) was used to measure the GIPCs protein concentrations. Protein samples (30 μg per lane) were separated on a 12% SDS-PAGE gel and subsequently transferred to a polyvinylidene fluoride (PVDF) membrane (Millipore Corporation, Burlington, MA, USA).

### 2.9. Total RNA Extraction and Real-Time Fluorescence Quantitative PCR

Total RNA was extracted from GIPCs in 12-well plates using 500 μL of TRIzol according to the company’s instructions (Takara, Shiga, Japan). Total RNA was then reverse-transcribed to cDNA using the RevertAid First Strand cDNA Synthesis Kit (Thermo Fisher Scientific, Waltham, MA, USA). Reverse transcription quantitative PCR (RT-qPCR) was used to detect target gene expression using the SYBR Green Master Mix (Takara, Kusatsu, Shiga, Japan). The specific primers used for RT-qPCR are listed in Table 1. The target gene expression level was calculated using the 2−^ΔΔCt^ method and normalized to UXT levels.

### 2.10. Dual-Luciferase Reporter Experiment

The *IRF7* and *ABCD1* promoter sequences were subcloned into the pGL3-basic plasmid. Then the luciferase reporter plasmid (1 μg) and Renilla luciferase plasmid (0.25 μg) were co-transfected into HEK293T cells using TurboFect Transfection Reagent (Thermo Fisher Scientific, Waltham, MA, USA). After 24 h post-transfection, firefly luciferase activity and Renilla luciferase activity were detected sequentially using the Dual-Luciferase Assay System (Promega, Madison, WI, USA). Firefly luciferase activity was normalized to Renilla luciferase activity to quantify relative promoter reporter activity.

### 2.11. RNA Sequencing (RNA-Seq) and Data Analysis

GIPCs were harvested 48 h after transfection with vector or HOXA5 (three biological replicates per group), and samples were submitted to Biomarker Technologies (Beijing, China) for RNA-seq. RNA concentration and purity were measured using a NanoDrop 2000, and integrity was assessed using an Agilent 2100 Bioanalyzer with the RNA 6000 Nano Kit. Libraries were prepared from 1 μg total RNA per sample using the NEBNext Ultra RNA Library Prep Kit for Illumina following poly(A)+ mRNA enrichment. Paired-end sequencing was performed on an Illumina platform, yielding at least 5.74 Gb of clean data per sample. Raw reads were filtered using in-house Perl scripts to remove adapter-containing, poly-N-containing, and low-quality reads. Clean reads were aligned to the *Capra hircus* reference genome CHIR_2.0 (GCA_000317765.2) with HISAT2, followed by transcript assembly and quantification using StringTie. Differential expression was analyzed using DESeq2, with *p* values < 0.05 and |foldchange| > 1.5 defining DEGs, and GO and KEGG enrichment analyses were conducted on the obtained DEGs. DEGs were selected randomly for RT-qPCR to validate the quality of RNA sequencing (primer information is listed in Table 1).

### 2.12. Statistical Analysis

Data were analyzed using GraphPad Prism 9.5 (Graphpad, San Diego, CA, USA). Comparisons between two groups were performed using t-tests, whereas multiple-group comparisons were analyzed using one-way ANOVA followed by Tukey multiple-comparison test. Data are expressed as the mean ± SEM. Statistical significance with * indicates *p* < 0.05, ** indicates *p* < 0.01. Each experiment was independently performed three times, with three biological replicates per treatment group in each experiment.

## 3. Results

### 3.1. HOXA5 Suppresses the Adipogenic Differentiation of GIPCs

To understand the role of HOXA5 in adipogenesis, the mRNA expression profiles of *HOXA5* in vitro were examined. Differentiation of GIPCs was induced with 50 μmol·L^−1^ oleic acid, and 0 d, 1 d and 5 d post-differentiation were chosen as the detection time points during lipogenesis. The results of Oil Red O staining showed that the content of lipid droplets gradually increased with prolonged induction, indicating the successful induction of GIPCs (Supplementary Figure S1A). The mRNA expression of *HOXA5* was significantly decreased during adipogenic differentiation compared to that in the 0-day cells (Supplementary Figure S1B). The results described above suggest that *HOXA5* may be an important gene that performs crucial functions in GIPC differentiation.

**Figure 1:**
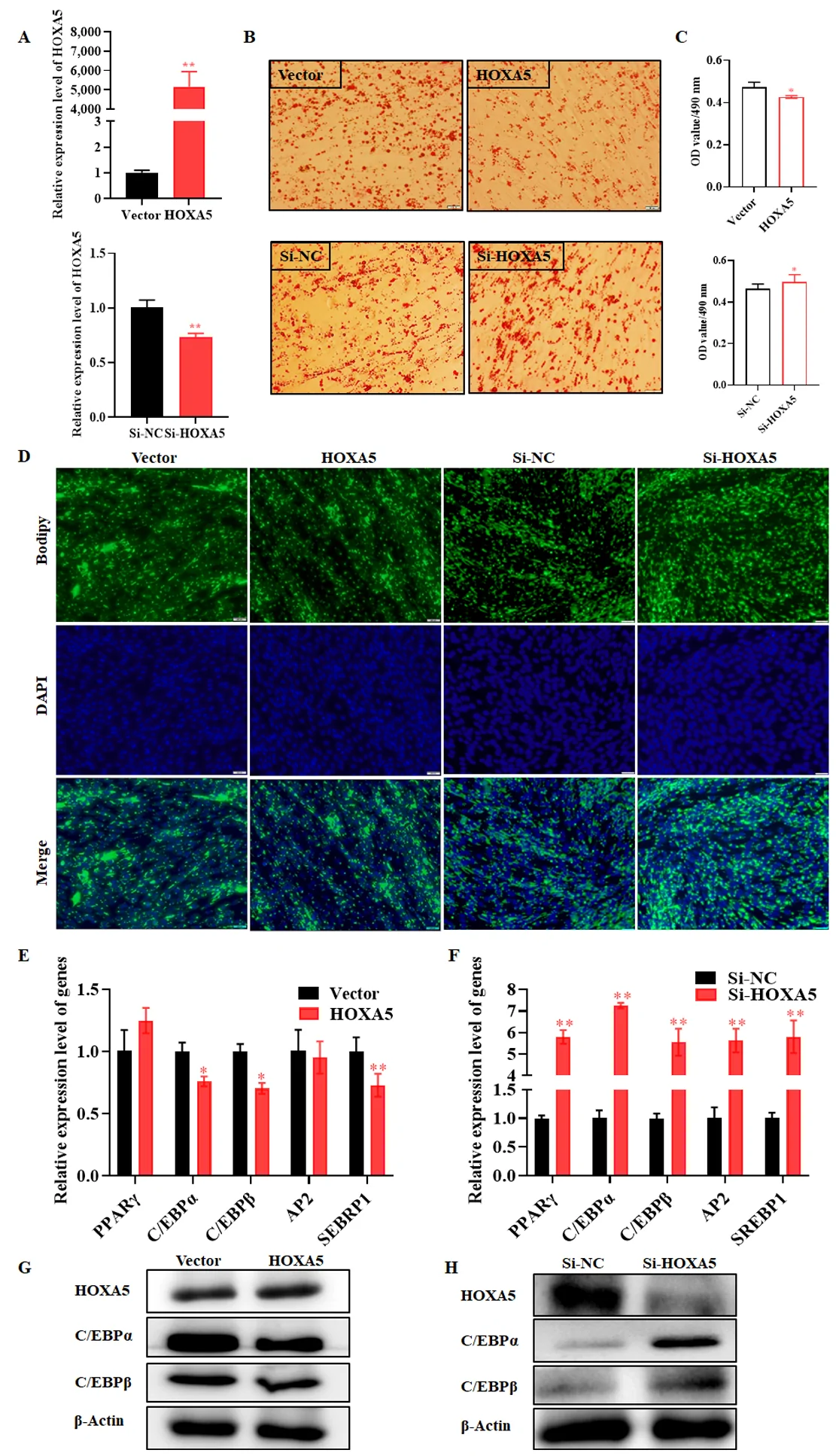
HOXA5 suppresses the adipogenic differentiation of GIPCs. (**A**) The mRNA expression of *HOXA*5 in GIPCs with HOXA5 overexpression or interference. (**B**) GIPCs were stained with Oil Red O at 48 h after the induction of differentiation following HOXA5 overexpression or interference. (**C**) Oil Red O was extracted with isopropyl alcohol and quantified by measuring the absorbance at 490 nm. (**D**) Bodipy (green) staining of lipid droplets and DAPI (blue) staining of nuclei in GIPCs that transfected with HOXA5 or HOXA5 interference.Staining was performed at 48 h after differentiation induction with oleic acid (50 μmol·L^−1^). Scale bar = 20 μm. (**E**,**F**) The mRNA expression of adipocyte differentiation marker genes in GIPCs that were overexpressed or knocked down HOXA5. (**G**,**H**) Protein expression of adipocyte marker genes in GIPCs with HOXA5 overexpression or interference was detected. Results are expressed as the means ± SEM, *n* = 3. * *p* < 0.05, ** *p* < 0.01.

To further examine the effect of HOXA5 on GIPCs adipogenesis, a HOXA5 overexpression plasmid (referred to hereafter as HOXA5) was constructed, using pEGFP-N1 as the control Vector (referred to as Vector henceforth). RNA interference and overexpression assays were performed in GIPCs. The mRNA and protein expression levels of HOXA5 were significantly increased in the HOXA5-overexpression group compared to the Vector group (Figure 1A,G). When compared to the cells transfected with Si-NC, the HOXA5 expression level in the Si-HOXA5-transfected cells was decreased (Figure 1A,H). Then, the effect of HOXA5 on GIPCs differentiation was evaluated by the formation of lipid droplets in GIPCs visualized by Oil Red O staining and Bodipy staining. Oil Red O staining (Figure 1B,C) and Bodipy staining (Figure 1D) results showed that overexpression of HOXA5 reduced the production of lipid droplets in GIPCs. However, a significant increase in the content of lipid droplets was observed in the HOXA5-interference group, contrasting with the result in the HOXA5-overexpression group. Then, the mRNA expression of differentiation marker genes was measured during the induction of GIPCs. Compared with the control group, the mRNA expression levels of *CEBP/α* (*p* < 0.05), *CEBP/β* (*p* < 0.05) and *SREBP1* (*p* < 0.01) were significantly suppressed in the overexpression group, whereas the expression of *PPARγ* and *AP2* did not change significantly (*p* > 0.05) (Figure 1E). Meanwhile, the expression of *PPARγ*, *CEBP/α*, *CEBP/β*, *AP2*, and *SREBP1* were significantly elevated (*p* < 0.01) in the interference group compared with the Si-NC group (Figure 1F). Through Western blot analysis, adipocyte differentiation factors, including CEBP/α and CEBP/β, were significantly downregulated when HOXA5 was overexpressed (Figure 1G) and significantly upregulated with HOXA5 interference (Figure 1H). The above results indicated that HOXA5 suppressed the adipogenic differentiation of GIPCs.

### 3.2. HOXA5 Promotes the Proliferation of GIPCs

**Figure 2:**
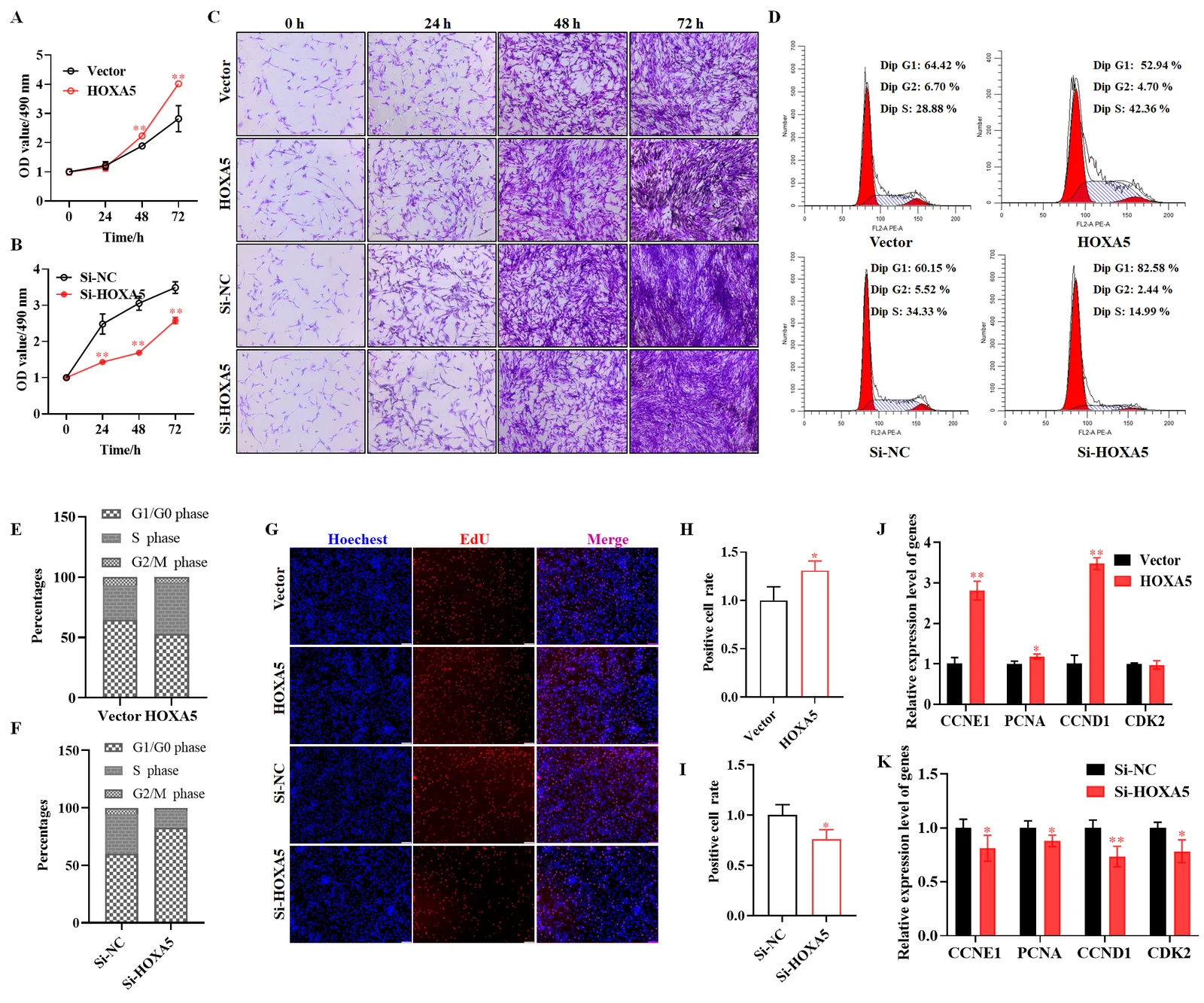
HOXA5 promotes the proliferation of GIPCs. (**A**,**B**) Cell proliferation was measured via MTT assay at 0, 24, 48 and 72 h for GIPCs after the overexpression of or interference with HOXA5. (**C**) Crystal violet staining was used to determine the proliferation of GIPCs at 0, 24, 48 and 72 h after transfection. (**D**–**F**) GIPCs were transfected with plasmids for HOXA5 expression or siRNA for HOXA5 silencing for 48 h, and cell cycle changes were determined by flow cytometry. The stacked bar charts in (**E**,**F**) show the percentages of cells in the G1/G0, S, and G2/M phases, with the different shaded patterns representing each cell cycle phase as indicated. (**G**) Cell proliferation was assessed at 48 h after the overexpression of and interference with HOXA5 using EdU. EdU-positive proliferating cells are shown in red, and nuclei were counterstained with Hoechst (blue). Scale bar = 10 μm. (**H**,**I**) The proliferation rate of GIPCs at 48 h after the overexpression of and interference with HOXA5 was measured. (**J**) Effect of the overexpression of HOXA5 on the expression of *CCNE1*, *PCNA*, *CCND1* and *CDK2*. (**K**) Effect of the interference of HOXA5 on the expression of *CCNE1*, *PCNA*, *CCND1* and *CDK2*. Results are expressed as the means ± SEM, *n* = 3. * *p* < 0.05, ** *p* < 0.01.

In addition to differentiation, the proliferation of preadipocytes can likewise affect fat deposition. To investigate the effects of HOXA5 on the proliferation of goat intramuscular preadipocytes, an MTT assay was performed, and the results revealed that HOXA5 overexpression significantly promoted GIPC proliferation at 48 and 72 h post-transfection (Figure 2A). In contrast, the proliferation of GIPCs was markedly suppressed in the HOXA5 interference group at different time points after transfection (24 h, 48 h and 72 h) (Figure 2B). Meanwhile, crystal violet staining confirmed the result. Overexpression of HOXA5 led to a notable increase in the cell count at 24 h, 48 h and 72 h. Conversely, knockdown of HOXA5 resulted in a marked decline in cell number at these same time points (Figure 2C). Then, cell cycle analysis revealed that HOXA5 overexpression significantly lowered the G1 phase proportion (64.42% to 52.94%) and notably increased the S phase proportion (28.88% to 42.36%) in GIPCs compared to the control group. However, upon knocking down HOXA5, the proportion of cells in the G1 phase significantly increased (60.15% to 82.58%), while the proportion in the S phase significantly decreased (34.33% to 14.99%) (Figure 2D–F). Furthermore, EdU staining results revealed that the EdU-positive rate in the HOXA5 overexpression group was significantly increased compared to the Vector group (Figure 2G,H). Conversely the EdU-positive rate of the HOXA5 interference group was significantly lower than that of the Si-NC group (Figure 2G,I). Then, the proliferation-related genes (*CCNE1*, *PCNA*, *CCND1*, and *CDK2*) were examined by RT-qPCR. Treatment with HOXA5 remarkably increased the mRNA levels of *CCNE1* (*p* < 0.01), *PCNA* (*p* < 0.05), and *CCND1* (*p* < 0.01), but the mRNA levels of *CDK2* were not altered (Figure 2J). Interestingly, the mRNA levels of *CCNE1* (*p* < 0.05), *PCNA* (*p* < 0.05), *CCND1* (*p* < 0.01), and *CDK2* (*p* < 0.05) were significantly downregulated with HOXA5 knockdown (Figure 2K). These findings suggest that HOXA5 promotes the proliferation of GIPCs.

### 3.3. HOXA5 Transcriptionally Activates the Expression of IRF7 and ABCD1

**Figure 3:**
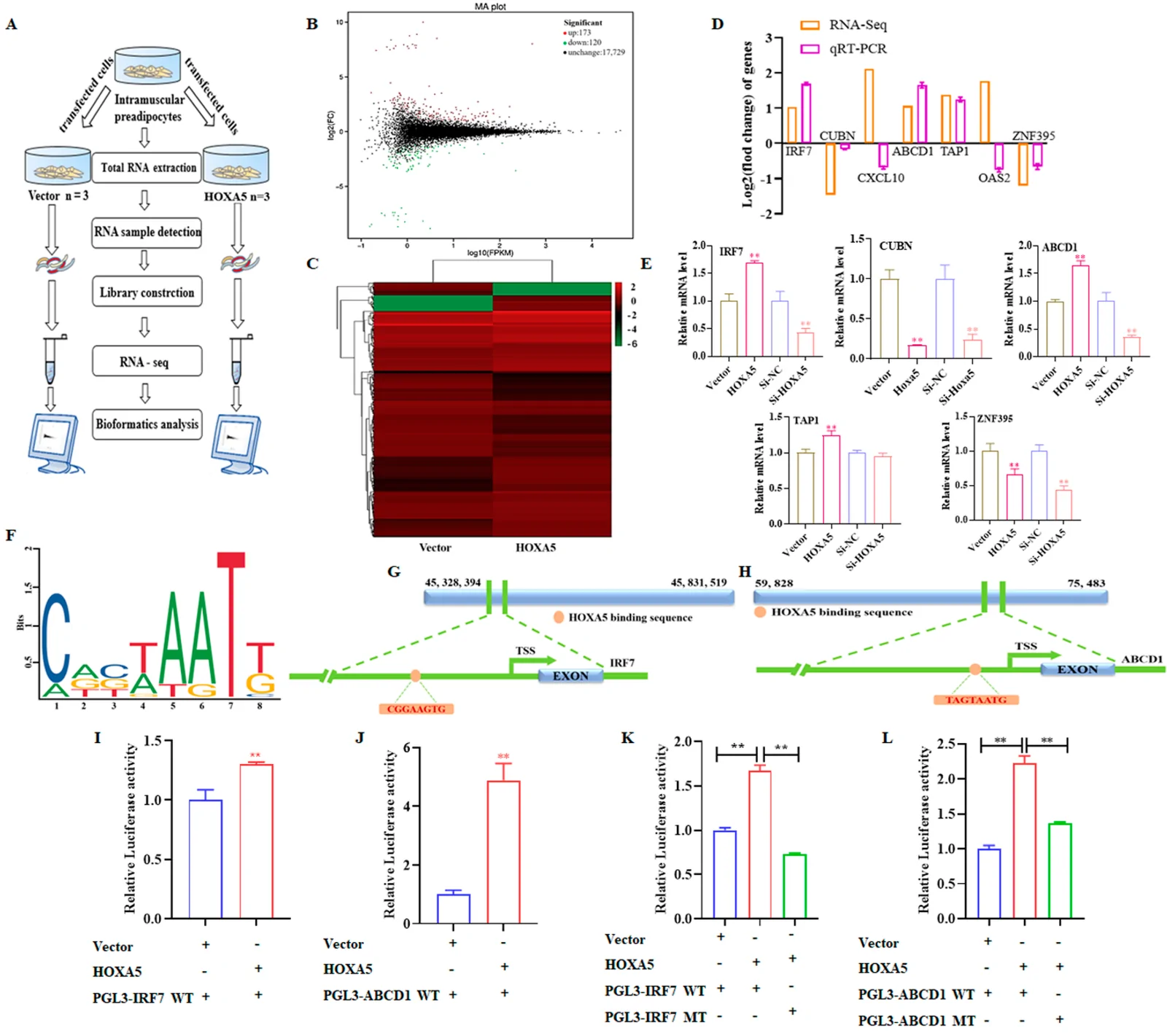
HOXA5 transcriptionally activates *IRF7* and *ABCD1*. (**A**) RNA-Seq workflow. (**B**,**C**) DEG gene expression is shown in the (**B**) volcano map and (**C**) heatmap, including 173 upregulated differential genes and 120 downregulated differentially expressed genes. (**D**) RT-qPCR verification results of differential genes in RNA-Seq data. (**E**) The mRNA levels of differential genes, including *IRF7*, *CUBN*, *ABCD1*, *TAP1* and *ZNF395* were detected after overexpression of or interference with HOXA5. (**F**–**H**) JASPAR (http://jaspar.genereg.net/), a transcription prediction web tool, was used to predict the binding sites of the transcription factor HOXA5 on the *IRF7* and *ABCD1* promoter regions. (**I**,**J**) The effects of HOXA5 on the transcriptional activation of *IRF7* and *ABCD1* determined by a dual-luciferase assay. (**K**,**L**) Dual-luciferase reporter assay evaluating the ratio of luciferase activity of WT-HOXA5 and MUT-HOXA5. Results are expressed as the means ± SEM, *n* = 3. ** *p* < 0.01.

In order to verify the molecular mechanism of HOXA5 in GIPC adipogenesis, RNA-seq was performed using samples harvested from GIPCs transfected with either HOXA5 or control Vectors for 48 h to identify downstream target genes (Figure 3A). In comparison to the Vector group, 293 DEGs (*p* value < 0.05, |foldchange| > 1.5) were identified in the HOXA5 overexpression group, of which 173 were upregulated and 120 were downregulated (Figure 3B,C). KEGG pathway enrichment analysis showed that the top 20 KEGG pathways were mostly related to immune and inflammatory responses (Supplementary Figure S2), such as the NOD-like receptor signaling pathway, RIG-I-like receptor signaling pathway, ABC transport pathway, TNF signaling pathway, and Toll-like receptor signaling pathway. Although the above signaling pathways are primarily associated with immunity and the inflammatory response, emerging evidence demonstrates that they are also involved in the proliferation and differentiation of adipocytes. Thus, it is speculated that the genes enriched in these pathways might also take part in the proliferation and differentiation of adipocytes. By analyzing the above signaling pathways, we found that *GBP1*, *OAS2*, *GBP5* and *IRF7* were mainly enriched in the NOD-like receptor signaling pathway. In addition, other DEGs, including *CXCL10*, *ABCD1*, *ABCA6*, *TAP1* and *CUBN* were enriched in other pathways (See Supplemental Table S1). Among these DEGs, genes potentially associated with adipocyte proliferation and differentiation were selected for validation using RT-qPCR. The expression trends of *IRF7*, *CUBN*, *ABCD1*, *TAP1* and *ZNF395* were consistent with the sequencing results, suggesting that the sequencing data were highly credible (Figure 3D).

Reportedly, HOXA5 as a transcription factor can exert biological functions by regulating the expression of downstream target genes. Therefore, it is likely that it regulates GIPC adipogenesis by changing the transcriptional activity of downstream target genes. We aimed to determine whether the expression levels of the genes related to lipid deposition verified above would show an opposite trend after interfering with HOXA5. The results showed that only the expression levels of *IRF7* and *ABCD1* demonstrated an inverse trend upon both overexpression of and interference with HOXA5 (Figure 3E). As such, the analyses above revealed that *IRF7* and *ABCD1* may be potential downstream targets of HOXA5. Subsequently, the JASPAR online tool (http://jaspar.genereg.net/) was employed to predict the binding sequences of HOXA5 within the promoter regions of *IRF7* and *ABCD1*, spanning from +100 to −2000 base pairs. Potential binding sequences for HOXA5 were identified on the promoters of *IRF7* and *ABCD1*, as illustrated in Figure 3F–H. To assess the transcriptional activity of HOXA5 on these two genes, dual luciferase reporter assays were conducted. The findings indicated that HOXA5 is capable of binding to the promoters of *IRF7* and *ABCD1*, thereby upregulating their expression, as depicted in Figure 3I,J. Furthermore, mutation of the HOXA5 binding site resulted in a significant reduction in luciferase activity in the mutant group compared to the wild-type group (Figure 3K,L). Collectively, these results confirm that HOXA5 specifically targets and regulates the expression of *IRF7* and *ABCD1*.

### 3.4. IRF7 Positively Regulates Differentiation and Proliferation in GIPCs

**Figure 4:**
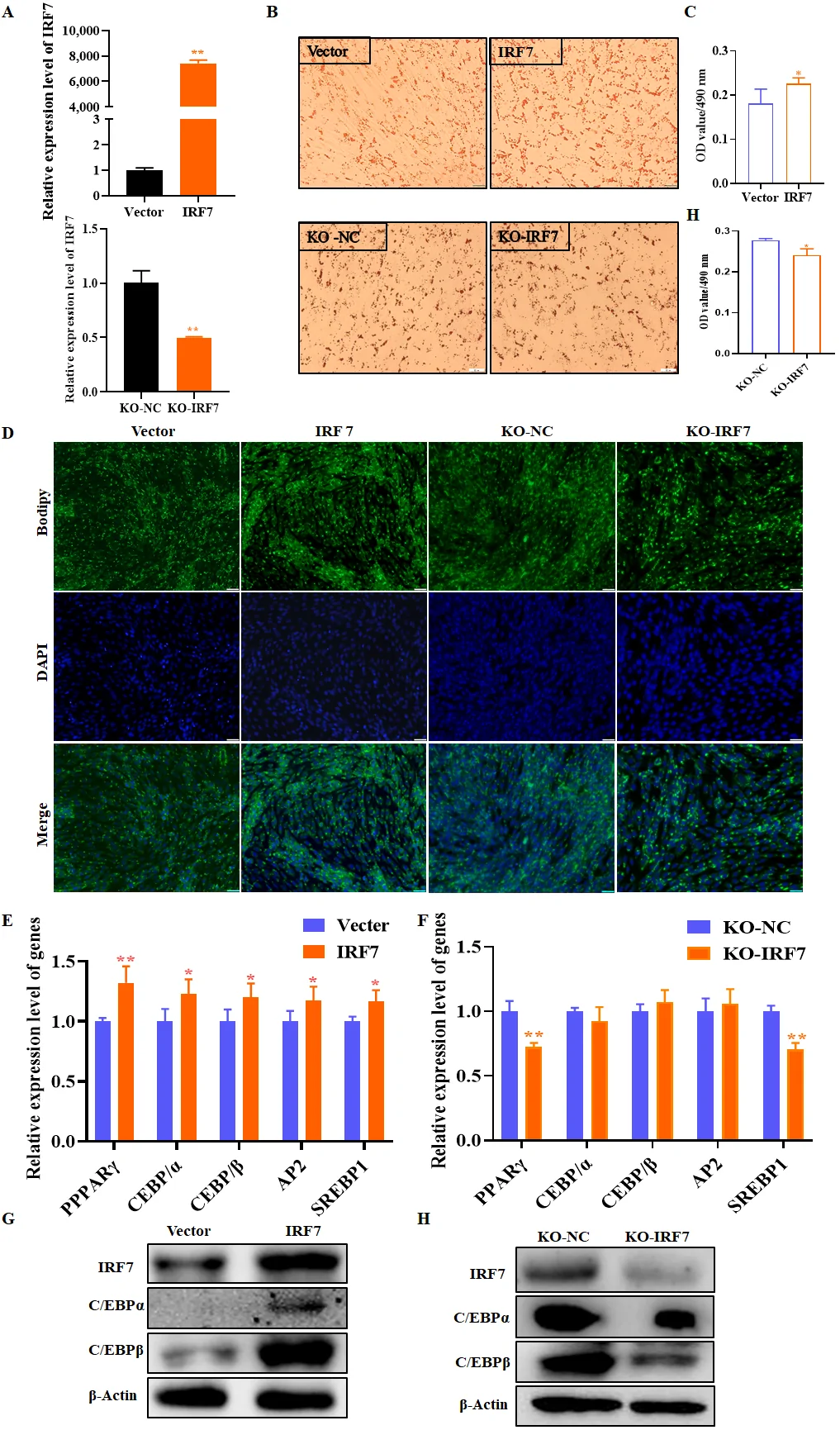
IRF7 positively regulates adipogenesis in GIPCs. (**A**) The mRNA expression of *IRF7* in GIPCs with IRF7 overexpression or knockout. (**B**) GIPCs were stained with Oil Red O at 48 h after the induction of differentiation when overexpressing or knocking out IRF7. (**C**) Oil Red O was extracted with isopropyl alcohol and quantified by measuring the absorbance at 490 nm. (**D**) Bodipy (green) staining of lipid droplets and DAPI (blue) staining of nuclei were performed for GIPCs transfected with IRF7 or with IRF7 knocked out. Staining was performed at 48 h after differentiation induction with oleic acid (50 μmol·L^−1^). Scale bar = 20 μm. (**E**,**F**) The mRNA expression of differentiated adipocyte marker genes in GIPCs with IRF7 overexpression or knockout. (**G**,**H**) The protein expression of adipocyte marker genes in GIPCs with IRF7 overexpression or knockout. Results were expressed as means ± SEM, n = 3. * *p* < 0.05, ** *p* < 0.01. Results are expressed as the means ± SEM, *n* = 3. * *p* < 0.05, ** *p* < 0.01.

Because *IRF7* is one of the downstream targets of HOXA5, we further explored its role in GIPC differentiation. An IRF7 expression plasmid (referred to hereafter as IRF7, with its control named Vector) and its knockout Cas9 plasmids (referred to hereafter as KO-IRF7, with its control named KO-NC) were constructed. Both the expression plasmid and knockout Vector were effective, GIPCs transfected with the IRF7 expression plasmid successfully upregulated its expression, while IRF7 knockout effectively downregulated its expression (Figure 4A,G,H). The Oil Red O and Bodipy staining results indicated that overexpression of IRF7 led to an increase in lipid droplets in GIPCs, whereas IRF7 knockout reduced lipid droplet accumulation compared to the control groups (Figure 4B–D). RT-qPCR and Western blot were used to detect the critical adipocyte differentiation markers, and the mRNA levels of *PPARγ*, *CEBP/α*, *CEBP/β*, *AP2*, and *SREBP1* were markedly promoted by IRF7 overexpression (Figure 4E). Also, the protein levels of CEBP/α and CEBP/β were significantly increased in the IRF7-overexpression cells (Figure 4G). Additionally, the mRNA levels of *PPARγ* and *SREBP1*, as well as the protein levels of CEBP/α and CEBP/β, which are associated with adipocyte differentiation, were significantly inhibited by IRF7 knockout (Figure 4F,H). In conclusion, these results indicated that IRF7 promotes the adipogenic capacity of GIPCs.

**Figure 5:**
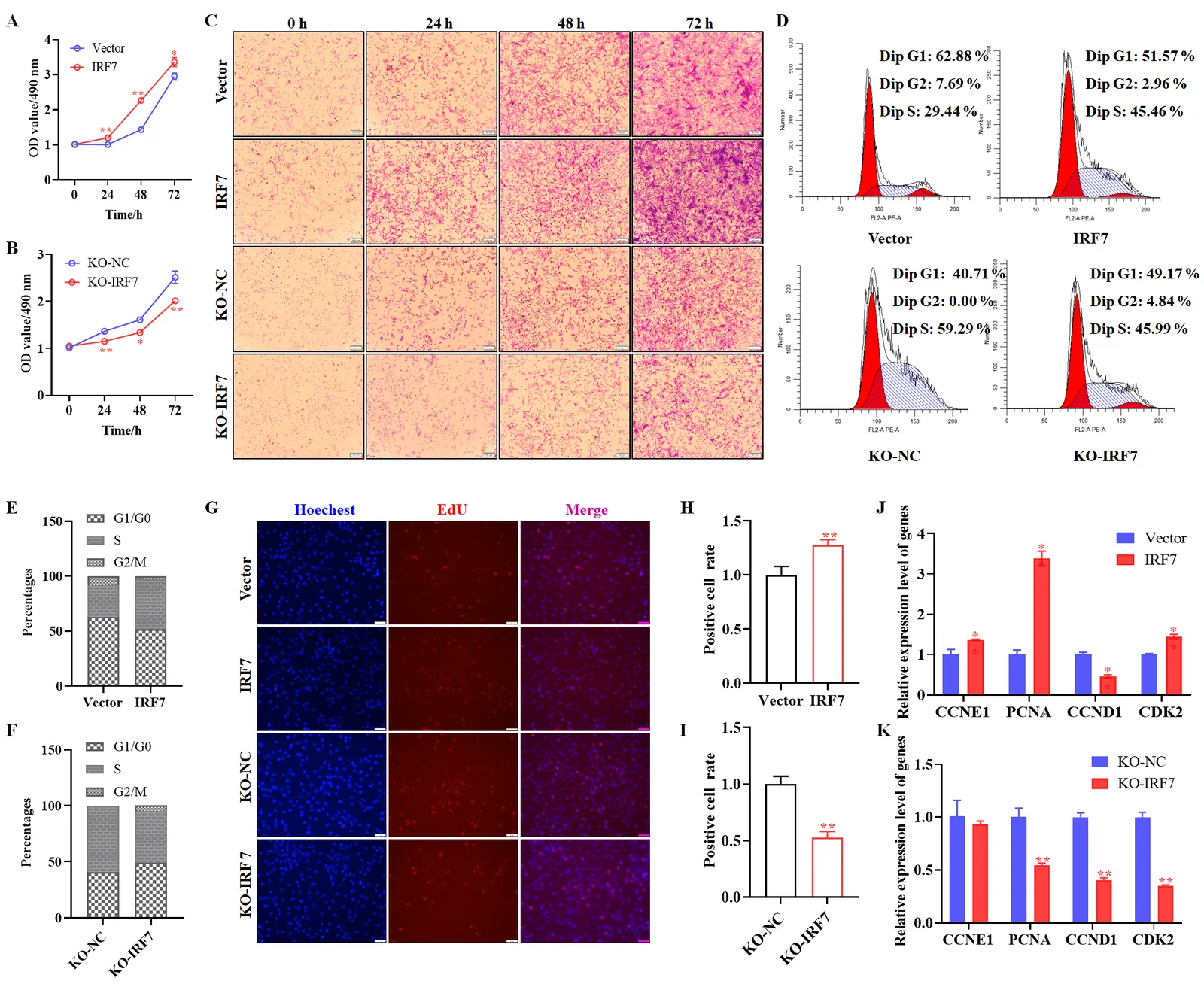
IRF7 positively regulates the proliferation of GIPCs. (**A**,**B**) Cell proliferation was measured via MTT assay at 0, 24, 48 and 72 h for GIPCs after overexpression or knockout of IRF7. (**C**) Crystal violet staining was used to determine the proliferation of GIPCs at 0, 24, 48 and 72 h after transfection. (**D**–**F**) GIPCs were transfected with IRF7-expressing plasmids or IRF7 knockout plasmids for 48 h, and cell cycle changes were determined by flow cytometry. The stacked bar charts in (**E**,**F**) show the percentages of cells in the G1/G0, S, and G2/M phases, with the different shaded patterns representing each cell cycle phase as indicated. (**G**) Cell proliferation was assessed at 48 h after the overexpression and knockout of IRF7 using EdU. EdU-positive proliferating cells are shown in red, and nuclei were counterstained with Hoechst (blue). Scale bar = 10 μm. (**H**,**I**) The proliferation rate of GIPCs at 48 h after the overexpression and knockout of IRF7 was measured. (**J**) Effect of the overexpression of IRF7 on the expression of *CCNE1*, *PCNA*, *CCND1* and *CDK2*. (**K**) Effect of the knockout of IRF7 on the expression of *CCNE1*, *PCNA*, *CCND1* and *CDK2*. Results are expressed as the means ± SEM, *n* = 3. * *p* < 0.05, ** *p* < 0.01.

To understand whether IRF7 was involved in GIPC proliferation, MTT assays and crystal violet staining were used to measure the activity and number of GIPCs at different times in culture. The results showed that IRF7 overexpression remarkably increased the number of GIPCs at 24 h, 48 h and 72 h post-transfection, whereas IRF7 knockout remarkably decreased the number of GIPCs (Figure 5A–C). Additionally, cell cycle analysis revealed that IRF7 overexpression significantly reduced the proportion of GIPCs in the G1 phase (62.88% to 51.57%) and significantly increased it in the S phase (29.44% to 45.46%), whereas IRF7 knockout had the opposite effect (Figure 5D–F). Likewise, EdU staining revealed that IRF7 overexpression significantly increased the percentage of EdU-positive cells (Figure 5G,H), while there were fewer cells positive for EdU amongst those with IRF7 knockout (Figure 5G,I). Moreover, the mRNA expression of marker genes closely responsible for proliferation (*CCNE1*, *PCNA*, and *CDK2*, except for *CCND1*), was markedly increased upon IRF7 overexpression (Figure 5J). Conversely, the mRNA expression of marker genes closely responsible for proliferation (*PCNA*, *CCND1*, and *CDK2*, except for *CCNE1*), was remarkably decreased by IRF7 knockout (Figure 5K). These results indicated that IRF7 facilitates the proliferation of GIPCs.

### 3.5. ABCD1 Suppresses the Adipogenic Differentiation and Proliferation of GIPCs

**Figure 6:**
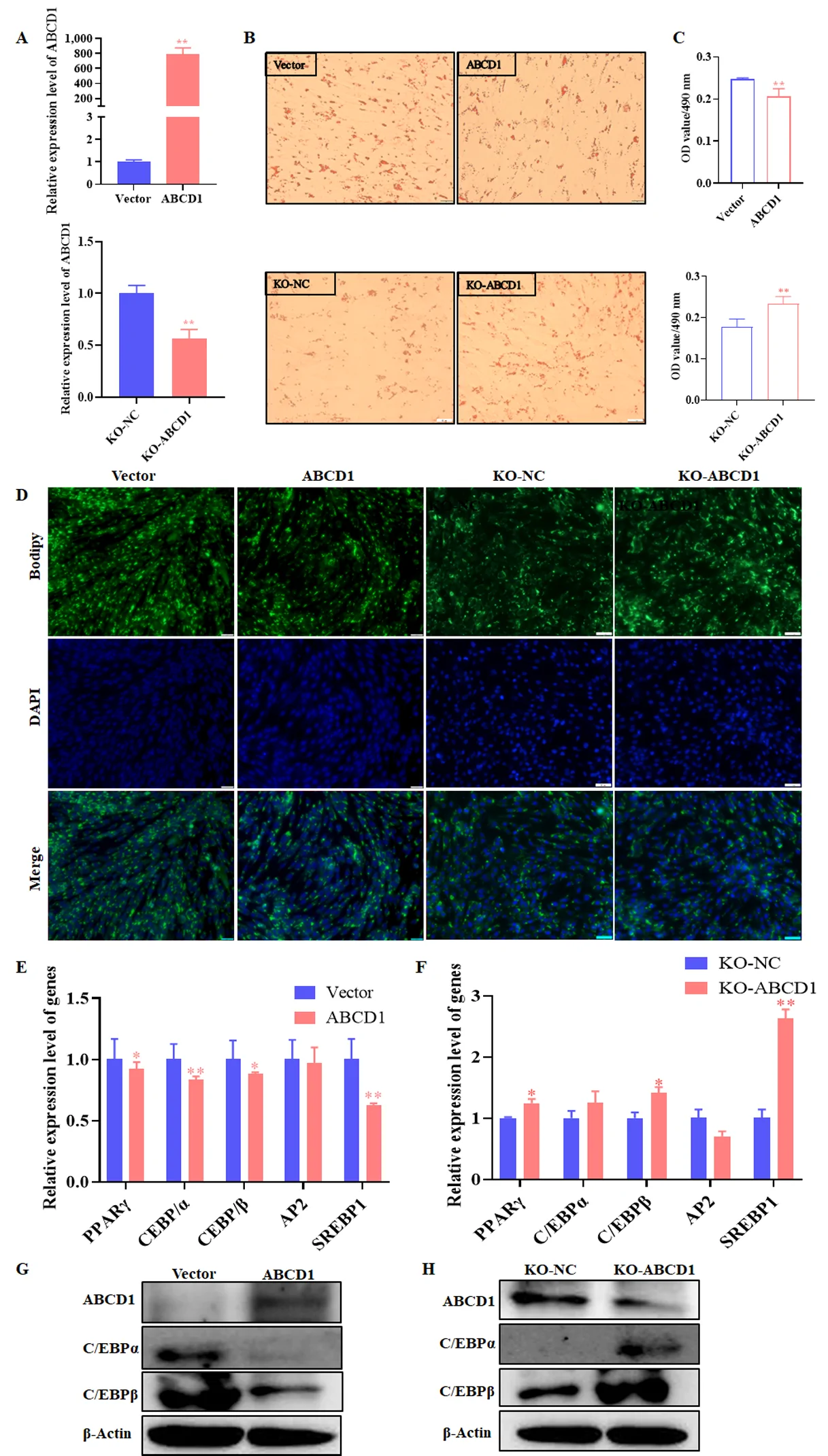
ABCD1 suppresses the adipogenic differentiation of GIPCs. (**A**) The mRNA expression of *ABCD1* in GIPCs with ABCD1 overexpression or knockout. (**B**) GIPCs were stained with Oil Red O at 48 h after the induction of differentiation when overexpressing or knocking out ABCD1. (**C**) Oil Red O was extracted with isopropyl alcohol and quantified by measuring the absorbance at 490 nm. (**D**) Bodipy (green) staining of lipid droplets and DAPI (blue) staining of nuclei were performed for GIPCs transfected with ABCD1 or with ABCD1 knocked out. Staining was performed at 48 h after differentiation induction with oleic acid (50 μmol·L^−1^). Scale bar = 20 μm. (**E**,**F**) The mRNA expression of differentiated adipocyte marker genes in GIPCs with ABCD1 overexpression or knockout. (**G**,**H**) The protein expression of adipocyte marker genes in GIPCs with ABCD1 overexpression or knockout was detected. Results are expressed as means ± SEM, n = 3. * *p* < 0.05, ** *p* < 0.01.

To investigate the role of ABCD1 in regulating the adipogenic differentiation of GIPCs, cells were transfected with either ABCD1 overexpression plasmids or ABCD1 knockout plasmids. The significant overexpression or knockout of ABCD1 in GIPCs was validated using RT-qPCR and Western blot analyses (Figure 6A,G,H). Subsequently, lipid droplet accumulation in differentiated GIPCs with either overexpression or knockout of ABCD1 was assessed using Oil Red O and Bodipy staining. The results of Oil Red O and Bodipy fluorescent staining indicated that overexpression of ABCD1 significantly reduced intracellular lipid droplet accumulation (Figure 6B–D). The intracellular lipid droplet accumulation in GIPCs was significantly higher in the ABCD1 knockout group compared to the control group (Figure 6B–D). Additionally, RT-qPCR results indicated that the mRNA levels of *PPARγ*, *CEBP/α*, *CEBP/β* and *SREBP1* were significantly reduced following ABCD1 overexpression (Figure 6E). Conversely, the mRNA levels of *PPARγ*, *CEBP/β* and *SREBP1* were significantly elevated after ABCD1 knockout. Western blot analysis revealed that overexpression of ABCD1 led to a significant decrease in the protein expression levels of CEBP/α and CEBP/β (Figure 6G). Conversely, knockout of ABCD1 resulted in a significant increase in the protein expression levels of CEBP/α and CEBP/β (Figure 6H). These findings suggest that ABCD1 plays a suppressive role in the adipogenic differentiation of GIPCs.

**Figure 7:**
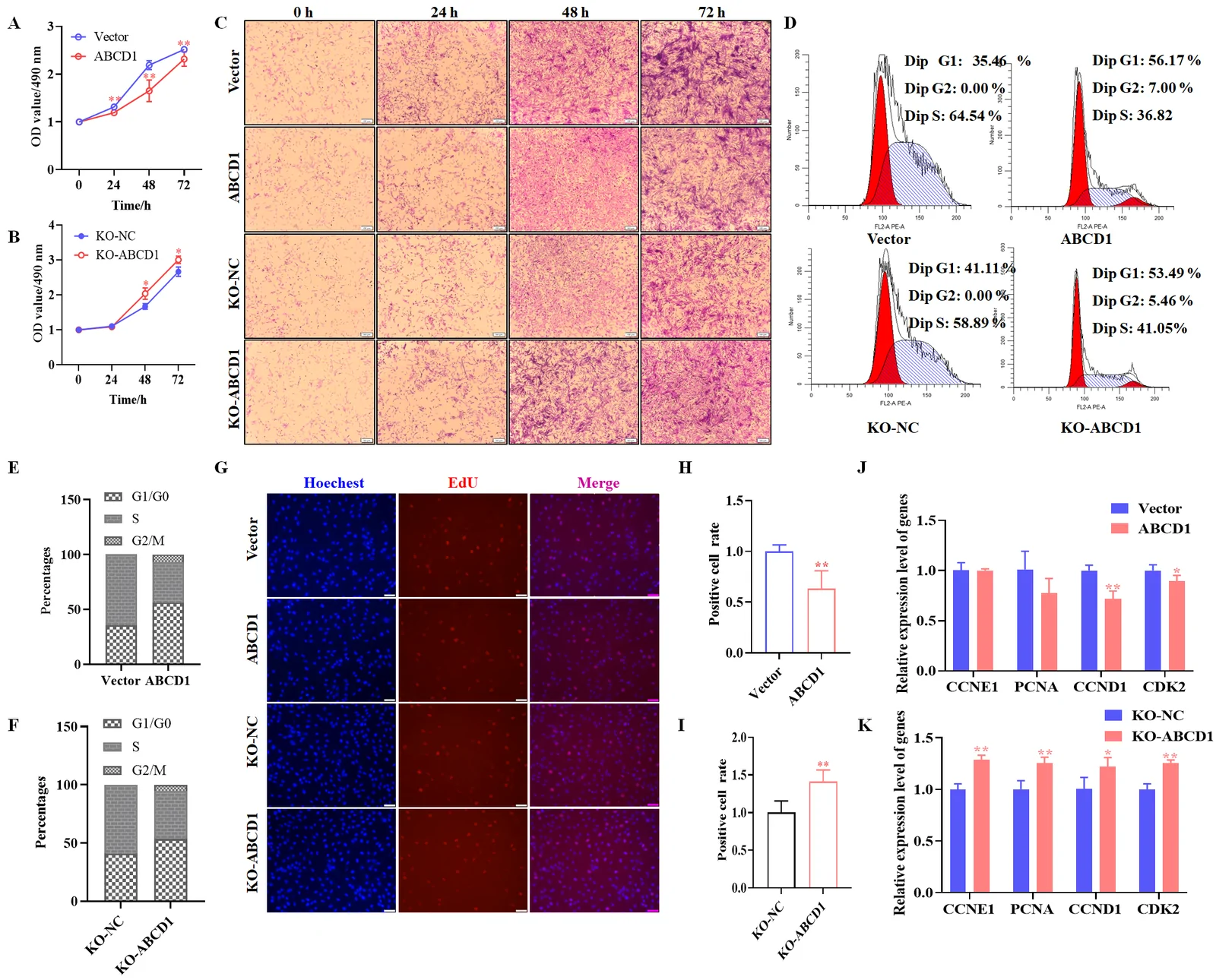
ABCD1 suppresses the proliferation of GIPCs. (**A**,**B**) Cell proliferation was measured via MTT assay at 0, 24, 48 and 72 h for GIPCs after the overexpression or knockout of ABCD1. (**C**) Crystal violet staining was used to determine the proliferation of GIPCs at 0, 24, 48 and 72 h after transfection. (**D**–**F**) GIPCs were transfected with ABCD1 expressing plasmids or ABCD1 knockout plasmids for 48 h, and cell cycle changes were determined by flow cytometry. The stacked bar charts in (E, F) show the percentages of cells in the G1/G0, S, and G2/M phases, with the different shaded patterns representing each cell cycle phase as indicated. (**G**) Cell proliferation was assessed at 48 h after the overexpression and knockout of ABCD1 using EdU. EdU-positive proliferating cells are shown in red, and nuclei were counterstained with Hoechst (blue). Scale bar = 10 μm. (**H**,**I**) The proliferation rate of GIPCs at 48 h after the overexpression and knockout of ABCD1 was measured. (**J**) Effect of the overexpression of ABCD1 on the expression of *CCNE1*, *PCNA*, *CCND1* and *CDK2*. (**K**) Effect of the knockout of ABCD1 on the expression of *CCNE1*, *PCNA*, *CCND1* and *CDK2*. Results are expressed as the means ± SEM, *n* = 3. * *p* < 0.05, ** *p* < 0.01.

To assess the regulatory function of ABCD1 in GIPCs, we conducted an analysis of proliferation in GIPCs with ABCD1 overexpression and knockout. The MTT assay demonstrated that compared with the Vector group, the ABCD1 overexpression group had a significantly lower number of cells at 24 h, 48 h and 72 h post-transfection (Figure 7A). In contrast, compared with the control group, the ABCD1 knockout group had a significantly higher number of cells at 48 h and 72 h post-transfection (Figure 7B). Meanwhile, crystal violet staining was also used to detect cell proliferation. Consistent with the MTT results, crystal violet staining images showed reduced staining in the ABCD1 overexpression group compared with the Vector group and increased staining in the ABCD1 knockout group compared with the KO-NC group (Figure 7C). Following ABCD1 overexpression, flow cytometry revealed an increased percentage of cells in the G1 phase and a decreased percentage in the S phase (Figure 7D,E). However, the results from ABCD1 knockout were similar to those observed with ABCD1 overexpression (Figure 7D,F). Moreover, the EdU assay demonstrated that the number of EdU-positive cells decreased in the ABCD1 overexpression group and was elevated in the ABCD1 knockout group (Figure 7G–I). In addition, the mRNA expression levels of cell proliferation markers, including *CCND1* and *CDK2* (excluding *CCNE1* and *PCNA*), were significantly reduced in the ABCD1 overexpression group relative to the Vector group (Figure 7J). Conversely, the mRNA expression levels of marker genes associated with cell proliferation, such as *CCNE1*, *PCNA*, *CCND1*, and *CDK2*, were significantly increased in the ABCD1 knockout group compared to the control group (Figure 7K). In conclusion, ABCD1 negatively regulates the proliferation and differentiation of GIPCs.

### 3.6. HOXA5 Promotes Proliferation and Inhibits Differentiation in GIPCs Through IRF7

**Figure 8:**
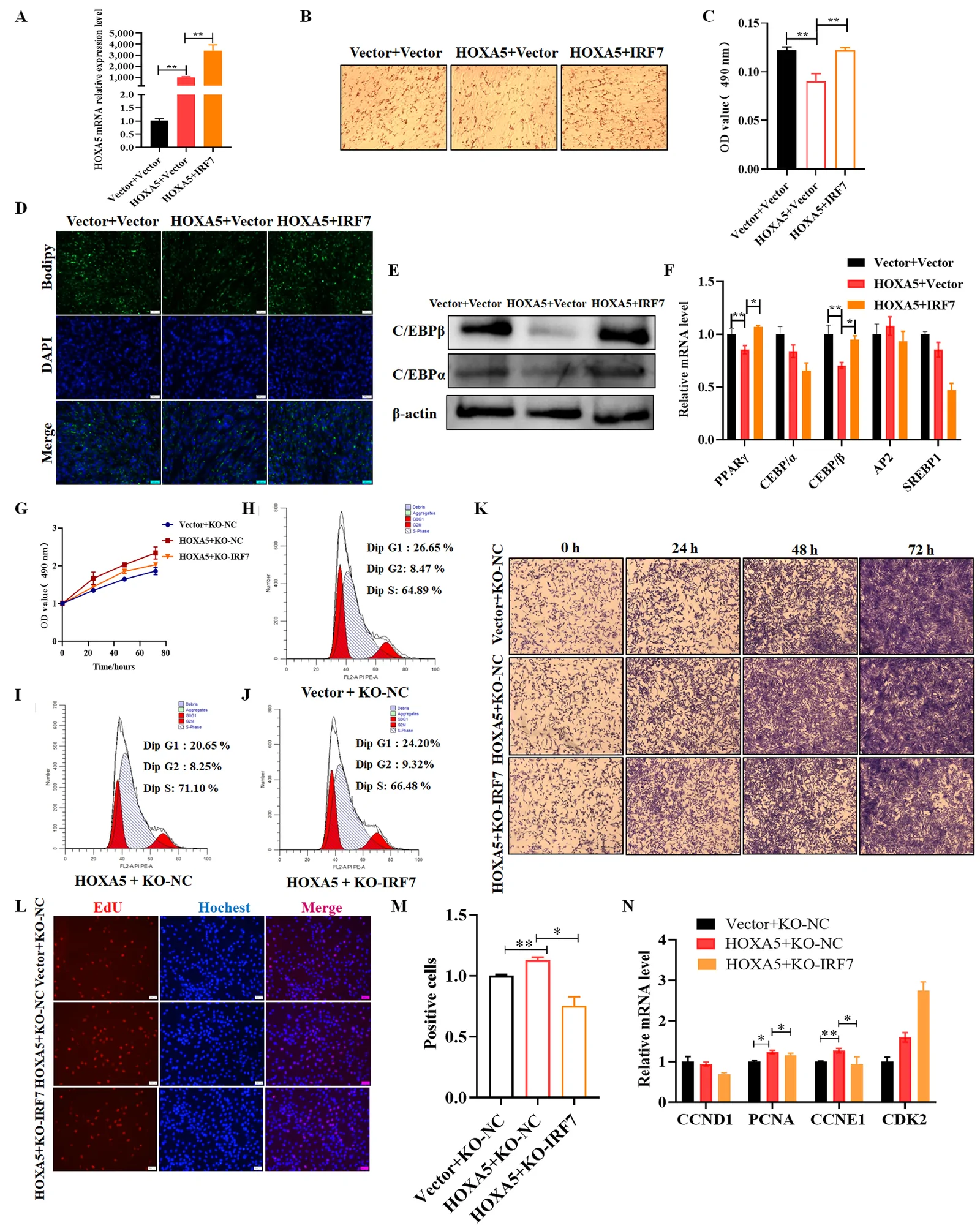
HOXA5 promotes proliferation and inhibits differentiation in GIPCs by targeting IRF7. (**A**) The mRNA expression of HOXA5 in GIPCs. (**B**) The images and (**C**) quantification of Oil red O staining in the Vector + Vector, HOXA5 + Vector, and HOXA5 + IRF7 groups (magnification: 400×, Scale bar = 20 μm). (**D**) The images of Bodipy (green) staining of lipid droplets in the Vector + Vector, HOXA5 + Vector, and HOXA5 + IRF7 groups (magnification: 400×, Scale bar = 20 μm). (**E**) The protein expression level of differentiation-related genes in the Vector + Vector, HOXA5 + Vector, and HOXA5 + IRF7 groups. (**F**) The mRNA expression levels of differentiation-related genes in the Vector + Vector, HOXA5 + Vector, and HOXA5 + IRF7 groups. (**G**–**K**) Cell proliferation was measured via MTT assay, flow cytometry and crystal violet staining in the Vector + KO-NC, HOXA5 + KO-NC, and HOXA5 + KO-IRF7 groups. (**L**,**M**) The proliferation of GIPCs was examined using Edu staining. (**N**) The mRNA expression levels of proliferation-related genes in Vector + KO-NC, HOXA5 + KO-NC, and HOXA5 + KO-IRF7 groups. Results are expressed as the means ± SEM, *n* = 3. * *p* < 0.05, ** *p* < 0.01.

To validate the role of HOXA5 in modulating GIPC proliferation and differentiation through IRF7, rescue experiments were performed. For adipogenic differentiation, we compared the Vector + Vector, HOXA5 + Vector, and HOXA5 + IRF7 groups to examine whether IRF7 overexpression attenuated HOXA5-induced inhibition of differentiation. Consistent with our hypothesis, Oil Red O and BODIPY staining showed that HOXA5 overexpression reduced lipid accumulation compared with the vector control, whereas IRF7 co-overexpression counteracted this reduction (Figure 8B–D). Consistently, IRF7 overexpression restored the HOXA5-induced downregulation of CEBP/α and CEBP/β protein levels (Figure 8E). In addition, HOXA5 overexpression reduced the mRNA levels of key differentiation-related genes, such as *PPARγ* and *CEBP/β*, a decrease that was also mitigated by IRF7 overexpression (Figure 8F).

For proliferation, we compared the Vector + KO-NC, HOXA5 + KO-NC, and HOXA5 + KO-IRF7 groups to determine whether IRF7 depletion counteracted HOXA5-induced proliferation. The results from MTT assays, crystal violet staining, flow cytometry and Edu staining indicated that HOXA5 overexpression significantly enhanced the proliferative capacity of GIPCs, but IRF7 depletion was able to mitigate this effect on GIPCs viability and number (Figure 8G–M). Furthermore, RT-qPCR analysis demonstrated that IRF7 depletion diminished the enhancing effects of HOXA5 overexpression on the expression of *PCNA* and *CCNE1* (Figure 8N). Collectively, these findings suggest that HOXA5 promotes proliferation and inhibits differentiation in GIPCs through IRF7.

### 3.7. HOXA5 Promotes Proliferation and Inhibits Differentiation in GIPCs Through ABCD1

**Figure 9:**
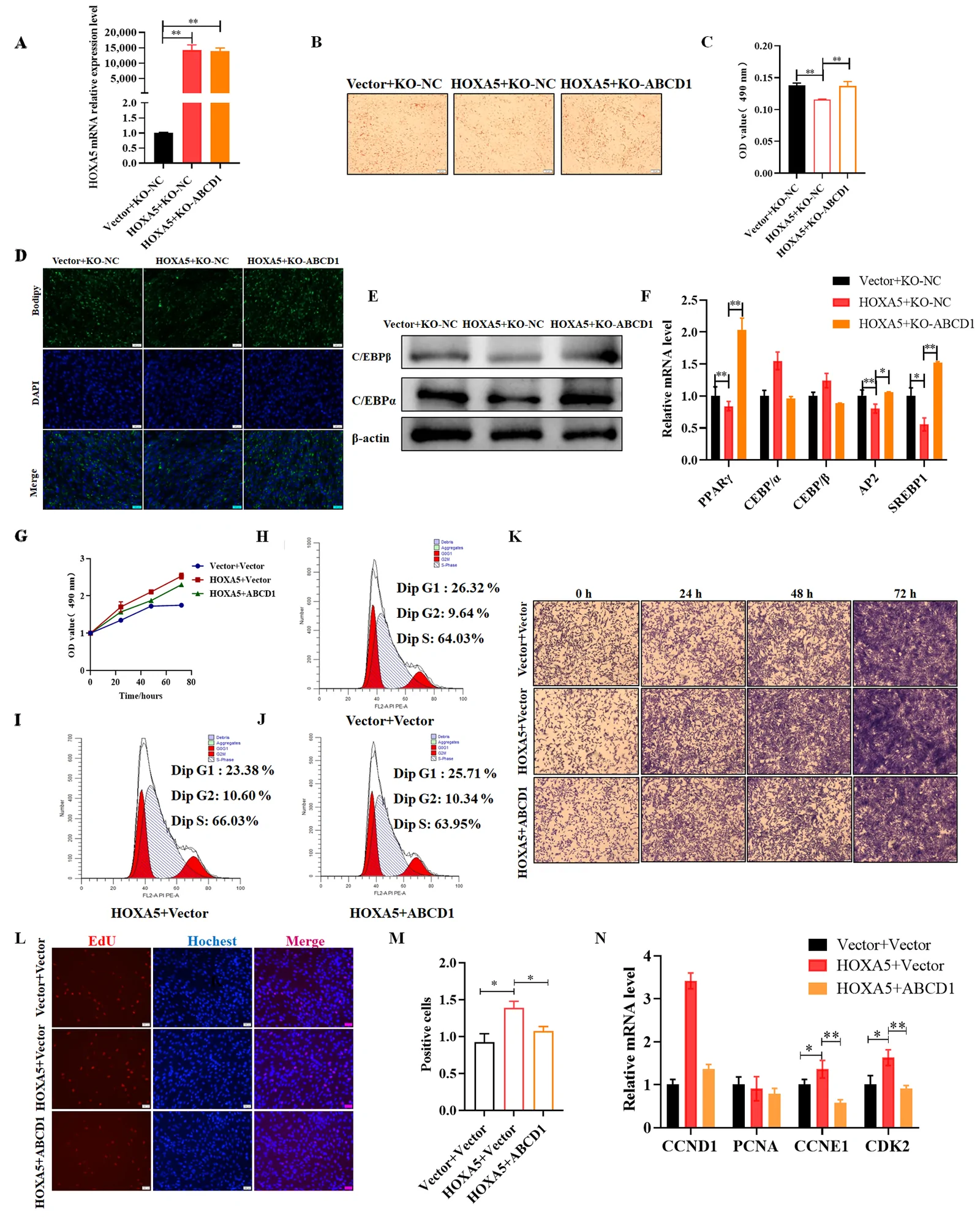
HOXA5 promotes proliferation and inhibits differentiation in GIPCs by targeting ABCD1. (**A**) HOXA5 expression in the Vector + KO-NC, HOXA5 + KO-NC, and HOXA5 + KO-ABCD1 groups at 48 h post-transfection. (**B**) The images and (**C**) quantification of Oil red O staining in the Vector + KO-NC, HOXA5 + KO-NC, and HOXA5 + KO-ABCD1 groups (magnification: 400×, Scale bar = 20 μm). (**D**) The images of Bodipy (green) staining of lipid droplets in the Vector + KO-NC, HOXA5 + KO-NC, and HOXA5 + KO-ABCD1 groups (magnification: 400×, Scale bar = 20 μm). (**E**,**F**) The protein and mRNA expression levels of differentiation-related genes in the Vector + KO-NC, HOXA5 + KO-NC, and HOXA5 + KO-ABCD1 groups. (**G**–**K**) Cell proliferation was measured via MTT assay, flow cytometry, and crystal violet staining in the Vector + Vector, HOXA5 + Vector, and HOXA5 + ABCD1 groups. (**L**) The proliferation of GIPCs was examined using EdU staining. (**M**,**N**) The mRNA expression level of proliferation-related genes in the Vector + Vector, HOXA5 + Vector, and HOXA5 + ABCD1 groups. Results are expressed as the means ± SEM, *n* = 3. * *p* < 0.05, ** *p* < 0.01.

To further investigate whether HOXA5 regulates the proliferation and adipogenic differentiation of goat intramuscular preadipocytes through ABCD1, we performed rescue experiments in HOXA5-overexpressing GIPCs. We first assessed differentiation in the Vector + KO-NC, HOXA5 + KO-NC, and HOXA5 + KO-ABCD1 groups. The HOXA5 + KO-NC group exhibited significantly lower lipid accumulation compared with the Vector + KO-NC group, as detected by Oil Red O and BODIPY staining. This reduction was alleviated after ABCD1 depletion in the HOXA5 + KO-ABCD1 group (Figure 9B–D). Consistently, ABCD1 depletion mitigated the HOXA5-induced decreases in the mRNA levels of *PPARγ*, *AP2 and SREBP1*, and the protein levels of C/EBPα and C/EBPβ (Figure 9E,F).

Next, we assessed proliferation in the Vector + Vector, HOXA5 + Vector, and HOXA5 + ABCD1 groups to examine whether ABCD1 overexpression counteracted HOXA5-induced proliferation. Compared with the Vector + Vector group, HOXA5 overexpression enhanced proliferation-related readouts, whereas ABCD1 co-overexpression attenuated these effects, as assessed by MTT assays, crystal violet staining, cell-cycle analysis, and EdU staining. The HOXA5-induced increases in CCNE1 and CDK2 mRNA levels were also diminished by ABCD1 co-overexpression. Together, these findings suggest that HOXA5 promotes proliferation and inhibits differentiation in GIPCs through ABCD1.

## 4. Discussion

IMF deposition has been demonstrated to be a critical determinant of meat quality in economically significant livestock [15,41,42]. Transcription factors, such as the KLF transcription factor family, have been implicated in the regulation of fat deposition, playing pivotal roles in this process [43,44,45]. The identification and characterization of key transcription factors and their regulatory networks are fundamental to enhancing meat quality in animals. Therefore, this study investigates the influence of HOXA5 on lipid deposition in goat intramuscular adipocytes, given that HOXA5 expression is downregulated during the differentiation of goat intramuscular adipocytes (Supplementary Figure S1A,B).

In our prior investigation, we observed that HOXA5 expression was altered during the differentiation of subcutaneous adipocytes in goats. HOXA5 was found to promote the differentiation of subcutaneous adipocytes while inhibiting the proliferation of subcutaneous preadipocytes [24]. Conversely, during the differentiation of intramuscular adipocytes, HOXA5 expression was downregulated. HOXA5 promotes proliferation and inhibits the differentiation of GIPCs. Two possibilities may help explain these contrasting phenomena. This is now supported by direct single-cell evidence in livestock species. In pigs, scRNA-seq revealed that subcutaneous fat is enriched with preadipocytes exhibiting an enhanced adipogenic potential, whereas intramuscular fat is characterized by a higher abundance of stem cells with lower adipogenic commitment [46]. In cattle, the intramuscular fat contains fewer adipose progenitor cells than subcutaneous fat, and these progenitors are adipogenically less committed and less differentiated [47]. These observations indicate that goat subcutaneous and intramuscular preadipocytes are unlikely to be equivalent cellular contexts. HOXA5 may therefore engage different transcriptional programs in the two progenitor pools, such that the same factor produces opposite net effects on proliferation and differentiation. Second, HOXA5 may act through different downstream mechanisms depending on cellular context. In human preadipocytes, HOXA5 represses WNT downstream genes *NFATC1* and *WNT2B* to maintain adipogenic differentiation [21]. In mouse adipocytes, HOXA5 promotes differentiation by activating *FABP4* and inhibiting PKA/HSL signaling [23]. In our study, IRF7 depletion attenuated HOXA5-induced proliferation, and ABCD1 depletion alleviated HOXA5-associated inhibition of differentiation. Whether these factors respond similarly in subcutaneous preadipocytes remains unknown.

To investigate the molecular mechanisms underlying HOXA5 regulation of lipid deposition in GIPCs, RNA-Seq was conducted following the overexpression of HOXA5. Through KEGG pathway analysis of 293 differentially expressed genes using RNA-Seq, it was found that genes such as *IRF7*, *CUBN*, *CXCL10*, *ABCD1*, *TAP1*, *OAS2*, and *ZNF395* were significantly enriched in pathways linked to immune responses and inflammation. Moreover, these pathways are also involved in the regulation of adipocyte proliferation and differentiation. Subsequent RT-qPCR validation demonstrated that the mRNA expression levels of *IRF7*, *CUBN*, *ABCD1*, *TAP1*, *OAS2*, and *ZNF395* were consistent with the RNA-Seq results, thereby confirming the accuracy of the sequencing data. Following the interference with HOXA5, the mRNA levels of *IRF7* and *ABCD1* exhibited an inverse relationship compared to the overexpression of HOXA5. HOXA5 is a multifunctional transcription factor that regulates various biological processes by targeting different genes. For instance, HOXA5 inhibits the proliferation of mouse adipocytes by transcriptionally regulating the downstream target gene CCNE1 [48]. But, HOXA5 reduces malignancy in breast epithelial cells by inhibiting NF-κB activity through the stabilization of its inhibitor IκBα [49]. Consequently, it is hypothesized that *IRF7* and *ABCD1* may be potential downstream target genes of HOXA5.

IRF7 is a transcription factor integral to the immune response and antiviral defense mechanisms [25,26,50,51]. Recent studies have elucidated that IRF7 is also implicated in energy metabolism. Specifically, an upregulation of *IRF7* mRNA levels has been observed in the white adipose tissue of obese mice. Conversely, mice with a whole-body knockout of IRF7 exhibit reduced adipose tissue mass under high-fat diet conditions, suggesting a pivotal role for IRF7 in adipogenesis [52]. Our findings indicated that IRF7 facilitates the proliferation and differentiation of IMF in goats, underscoring its significance in lipid metabolism and fat formation.

The *ABCD1* gene encodes a crucial cell membrane protein that plays a significant role in the transport and metabolism of fatty acids [53,54]. It facilitates the beta-oxidation of fatty acids within the peroxisome, primarily through the transport of long-chain fatty acids into the cellular peroxisome [55,56]. Our research demonstrated that ABCD1 inhibited the proliferation and differentiation of GIPCs. However, it remains unclear whether ABCD1 also influences lipid deposition in GIPCs by transferring long-chain fatty acids to peroxisomes for fatty acid β-oxidation.

**Figure 10:**
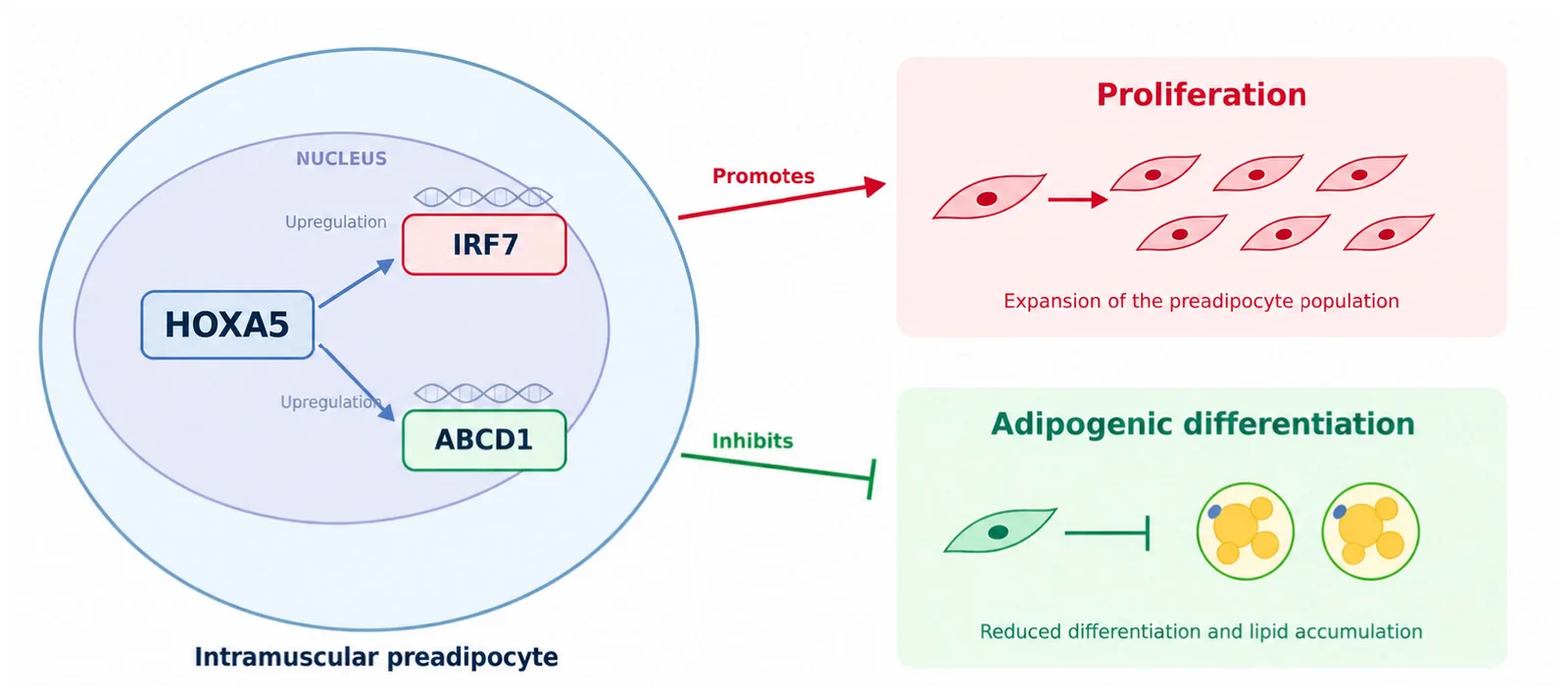
Schematic illustration of the proposed HOXA5 regulatory model in goat intramuscular preadipocytes. HOXA5 promotes proliferation and inhibits adipogenic differentiation via IRF7 and ABCD1. Arrows indicate promotion; T-shaped lines indicate inhibition.

This study investigated the regulatory role of HOXA5 in lipid deposition within GIPCs. The findings indicated that HOXA5 transcriptionally activates IRF7 and ABCD1, subsequently promoting adipocyte proliferation while inhibiting adipocyte differentiation. Based on these findings, we developed a schematic model illustrating the regulatory effects of HOXA5 on the proliferation and adipogenic differentiation of goat intramuscular preadipocytes (Figure 10). Nonetheless, further research is required to elucidate whether HOXA5 influences lipid deposition through specific signaling pathways.

## 5. Conclusions

IRF7 is a positive regulator of the proliferation and differentiation of intramuscular preadipocytes in goats, while ABCD1 is a negative regulator of these processes. In conclusion, HOXA5 not only targets the positive regulatory factor IRF7 that positively regulates proliferation and adipogenic differentiation, but also the negative regulatory factor ABCD1 that negatively regulates proliferation and differentiation. It has a unique dual regulatory effect on the proliferation and adipogenic differentiation of intramuscular adipocytes in goats but ultimately shows an inhibitory effect on intramuscular fat deposition. Our research, which identified the transcription factor HOXA5 as a regulator of lipid formation and its downstream target genes, offers novel insights into the molecular breeding of goats.

## Figures and Tables

**Table 1:** Primer information for quantitative real-time PCR (RT-qPCR).

Gene Name	Forward Sequence (5′–3′)	Reverse Sequence (5′–3′)
HOXA5	CTCATTTTGCGGTCGCTATC	ACGCTGAGATCCATGCCATT
CCNE1	CTCCCTGATTCCCACACCTG	CATAAGATGCTTGTCCCTCA
PCNA	AGTGGAGAACTTGGAAAGGAA	GAGACAGTGGAGTGGCTTTTGT
CCND1	TGAACTACCTGGACCGCT	CAGGTTCCACTTGAGTTTGT
CDK2	GCCAGGAGTTACTTCTATGC	TGGAAGAAAGGGTGAGCC
C/EBPα	CTCCGGATCTCAAGACTGCC	CCCCTCATCTTAGACGCACC
C/EBPβ	CCGCCTTTAAATCCATGGAA	CTCGTGCTCTCCGATGCTAC
PPARγ	AAGCGTCAGGGTTCCACTATG	GAACCTGATGGCGTTATGAGAC
AP2	GTCCTTCAAATGGGCCAGGA	CTGGTGGTAGTGACACCGTT
SREBP1	AACATCTGTTGGAGCGAGCA	TCCAGCCATATCCGAACAGC
IRF7	AAGCAGATTCGCGCATTTTT	ATGAGGGTCGGTAGGGTCCC
ABCD1	CTGTGGCCCACATATGGTGG	CGTCTTTCCAGTCGCACACG
CUBN	CAATAGCGTCCTTCATTCGT	TTGGAATCAAGCTGATGGATT
CXCL10	AATCCTGCCCACGTGTCGAG	CTCTTTCCGTGTTCGAGGAG
TAP1	TCTCGGTCAGTGCTTTTCAT	CAATAGCGTCCTTCATTCGT
OAS2	GAACCCTGCCAGTCCCTCAC	TCACAGTGCTTCATCCGTGC
ZNF395	ATGGAGATACCATACTCCCTGAT	ATCTTCTAGGGCCTCTGAGAATC
UXT	GCAAGTGGATTTGGGCTGTAAC	ATGGAGTCCTTGGTGAGGTTGT

## Data Availability

Data or models are not deposited in an official repository but are available from the authors upon reasonable request.

## Supplementary Materials

The following supporting information can be downloaded at: https://www.mdpi.com/article/10.3390/ani16213537/s1, Figure S1: HOXA5 expression significantly changed during goat intramuscular adipocyte differentiation. (A) Images of Oil red O staining at 0, 1, and 5 d after intramuscular preadipocytes adipogenic differentiation. (B) The expression level of ABCD1 during intramuscular preadipocyte differentiation. The mRNA level of HOXA5 at 0, 1, and 5 d after intramuscular preadipocytes adipogenic differentiation. * *p* < 0.05; ** *p* < 0.01; Figure S2: KEGG analysis of DE genes in the Vector vs. HOXA5 groups. KEGG enrichment pathway annotated classification results; Table S1: Differential genes enriched in signaling pathways related to lipid deposition.
