# Supplementary material for: HOXA5 Targets ABCD1 and IRF7 to Regulate the Proliferation and Differentiation of Goat Intramuscular Preadipocytes
Source: Animals (Basel). 2026 Nov 9;16(21):3537. doi: 10.3390/ani16213537 (PMC13761576; doi:10.3390/ani16213537)
Supplement: Supplementary file 1 [file animals-16-03537-s001.zip › animals-4588208-supplementary.pdf]

Supplementary Material

Supplementary Figure

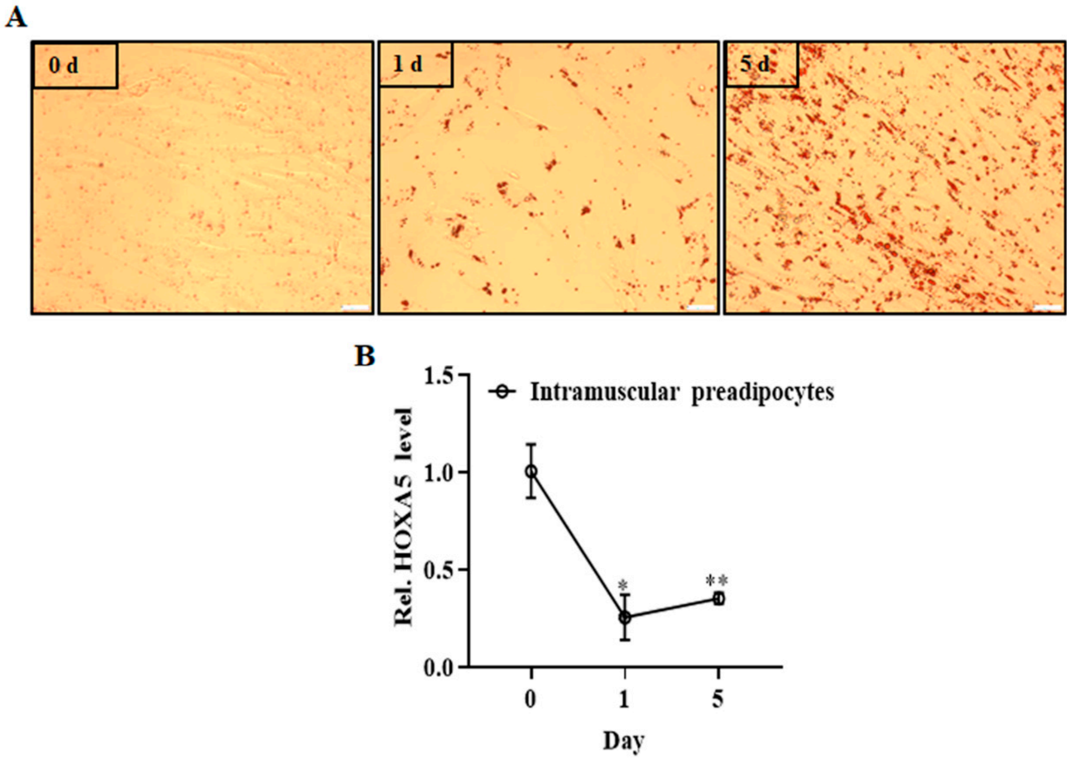

Supplementary Figure S1. HOXA5 expression significantly changed during goat intramuscular adipocyte differentiation. (A) Images of Oil red O staining at 0, 1, and 5 d after intramuscular preadipocytes adipogenic differentiation. (B) The expression level of ABCD1 during intramuscular preadipocyte differentiation. The mRNA level of HOXA5 at 0, 1, and 5 d after intramuscular preadipocytes adipogenic differentiation. * $p < 0.05$; ** $p < 0.01$.

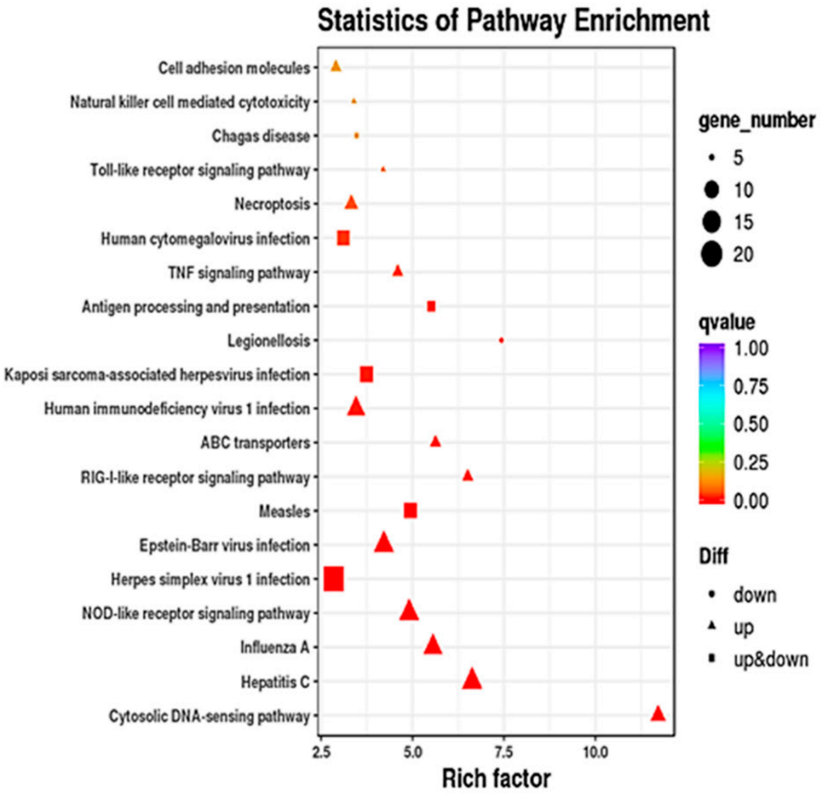

Supplementary Figure S2. KEGG analysis of DE genes in the Vector vs. HOXA5 groups. KEGG enrichment pathway annotated classification results.

Supplemental Table

Supplementary Table S1. Differential genes enriched in signaling pathways related to lipid deposition.

Pathway	gene-number	Contained
NOD-like receptor signaling pathway	12	GBP1, OAS2, GBP5, IRF7,
RIG-I-like receptor signaling pathway	6	CXCL10 and IRF7
ABC transporters	6	ABCD1, ABCA6 and TAP1
TNF signaling pathway	6	CXCL10, CUBN
Toll-like receptor signaling pathway	5	CXCL10, IRF7 and ZNF395

Note: The number of genes is the total number of genes enriched, including unannotated new genes, and only annotated genes are given in the table1
